# Supplementary material for: Causal Effect of the Tokyo 2020 Olympic and Paralympic Games on the Number of COVID-19 Cases under COVID-19 Pandemic: An Ecological Study Using the Synthetic Control Method
Source: J Pers Med. 2022 Feb 3;12(2):209. doi: 10.3390/jpm12020209 (PMC8879008; doi:10.3390/jpm12020209)
Supplement: Supplementary file 1 [file jpm-12-00209-s001.zip › Supplemental Table 1.pdf]

Supplementary Table 1. Post-RMSPE-to-pre-RMSPE ratio determined using synthetic control weight or regression weight

Prefecture	Synthetic control weight				Regression weight		
	Pre-RMSPE	Post-RMSPE	Post/Pre ratio	Rank	Pre-RMSPE	Post-RMSPE	Rank
Mie	0.207	4.941	23.837	1	0	4.602	6
Aichi	0.337	5.381	15.951	2	0	4.270	7
Kumamoto	0.177	2.218	12.534	3	0	2.478	18
Shiga	0.371	3.893	10.498	4	0	2.557	16
Ishikawa	0.513	4.628	9.023	5	0	7.048	3
Yamaguchi	0.327	2.390	7.313	6	0	1.534	26
Kyoto	0.284	1.866	6.567	7	0	3.284	10
Fukuoka	0.625	4.072	6.514	8	0	2.808	14
Kagoshima	0.357	2.120	5.946	9	0	0.993	30
Hiroshima	0.579	3.103	5.362	10	0	2.480	17
Gifu	0.324	1.667	5.149	11	0	1.843	21
Oita	0.546	2.683	4.919	12	0	1.122	28
Saga	0.486	2.084	4.285	13	0	4.888	5
Hyogo	0.273	1.155	4.228	14	0	1.927	20
Tokyo	1.919	7.866	4.100	15	0	7.412	2
Nara	0.359	1.427	3.975	16	0	1.471	27
Kochi	0.607	2.325	3.831	17	0	4.165	8
Wakayama	0.274	1.005	3.670	18	0	2.252	19
Okinawa	4.853	17.728	3.653	19	0	17.211	1
Tokushima	0.433	1.552	3.585	20	0	0.994	29
Ehime	0.419	1.464	3.497	21	0	6.521	4
Okayama	0.487	1.641	3.371	22	0	1.815	22
Toyama	0.446	1.373	3.080	23	0	2.722	15
Miyazaki	0.310	0.935	3.015	24	0	1.705	24
Nagasaki	0.403	1.167	2.897	25	0	2.889	12
Shimane	0.408	1.116	2.732	26	0	1.766	23
Osaka	1.956	4.313	2.205	27	0	2.912	11
Tottori	0.509	1.079	2.119	28	0	2.856	13
Kagawa	0.443	0.880	1.986	29	0	1.669	25
Fukui	0.699	1.222	1.750	30	0	3.395	9

RMSPE: root mean squared prediction error; Pre- and Post- indicate the RMSPEs before and after the opening of the Tokyo 2020 Olympic and Paralympic Games, respectively.
