# Supplementary figures and images for: Causal Effect of the Tokyo 2020 Olympic and Paralympic Games on the Number of COVID-19 Cases under COVID-19 Pandemic: An Ecological Study Using the Synthetic Control Method
Source: J Pers Med. 2022 Feb 3;12(2):209. doi: 10.3390/jpm12020209 (PMC8879008; doi:10.3390/jpm12020209)

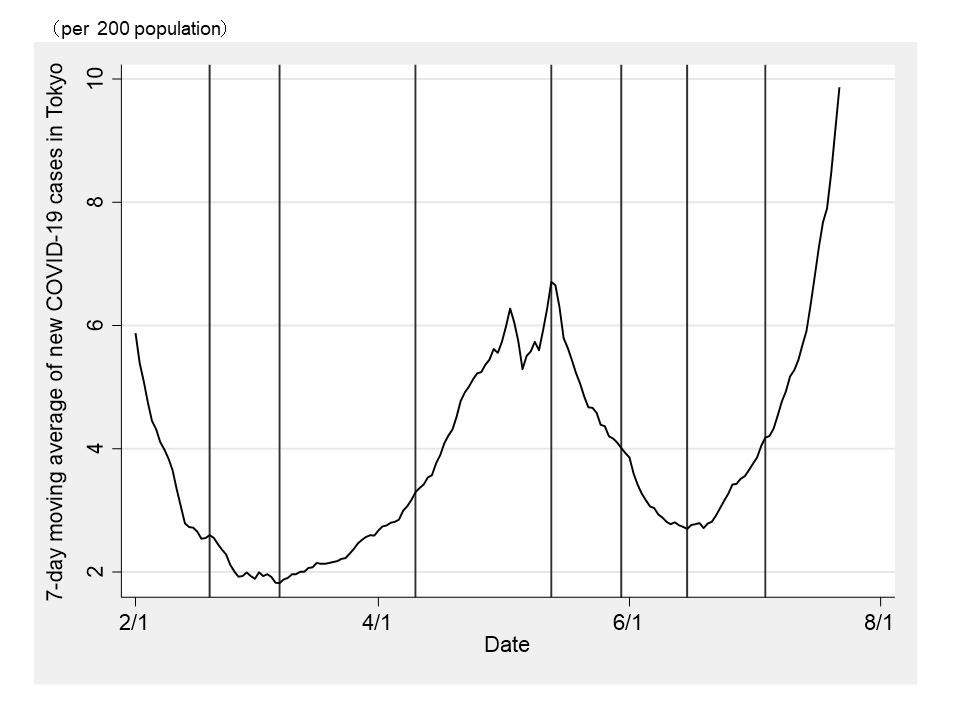

Supplement: Supplementary file 1 [file jpm-12-00209-s001.zip › Supplemental Figure 1.tif]

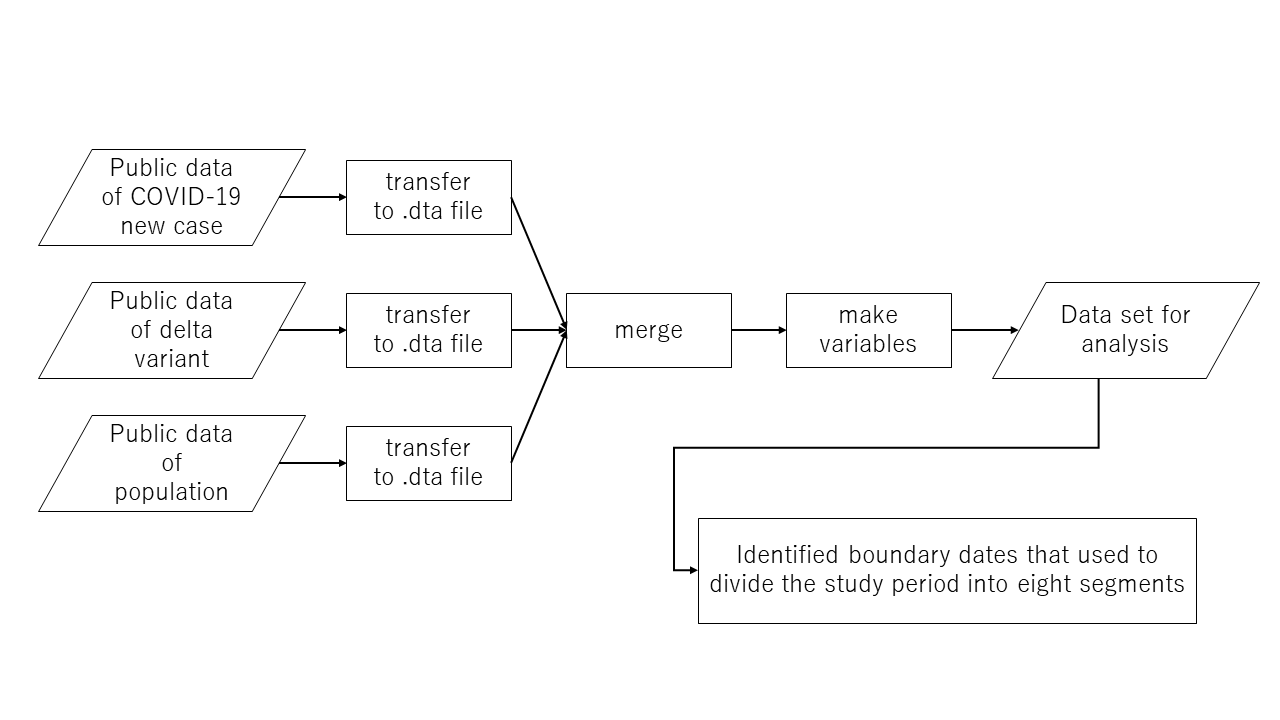

Supplement: Supplementary file 1 [file jpm-12-00209-s001.zip › Supplemental Figure 2.tif]

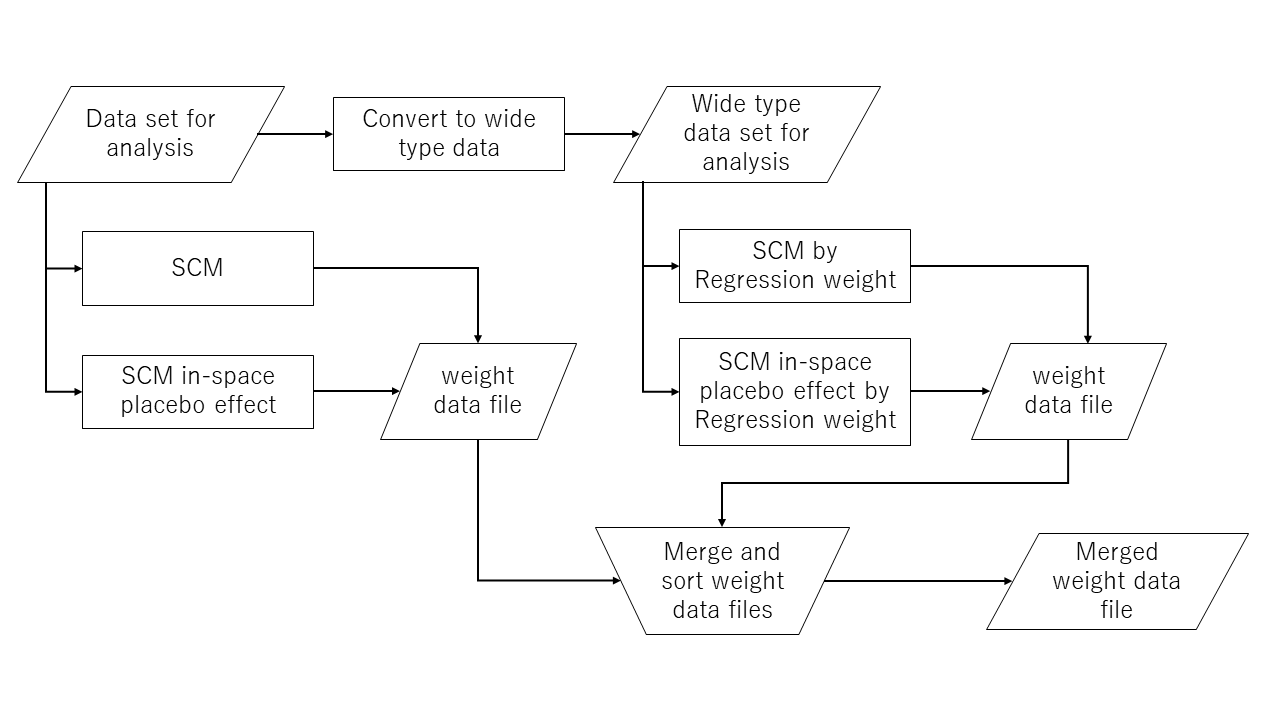

Supplement: Supplementary file 1 [file jpm-12-00209-s001.zip › Supplemental Figure 3.tif]

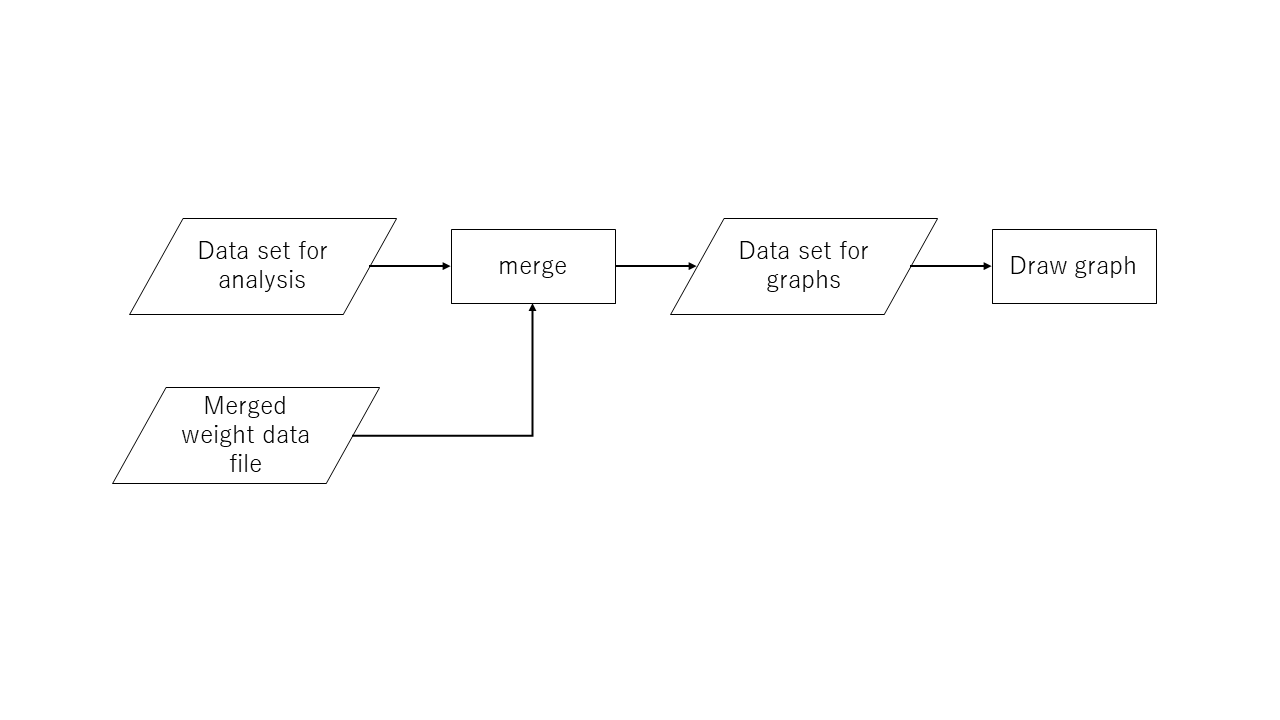

Supplement: Supplementary file 1 [file jpm-12-00209-s001.zip › Supplemental Figure 4.tif]
